# Differentiated regulation and health spending growth: European evidence from the new SCORE index

**DOI:** 10.3389/frhs.2026.1888179

**Published:** 2026-08-12

**Authors:** Emanuele Bracco

**Affiliations:** Department of Economics, University of Verona, Verona, Italy

**Keywords:** alcohol policy, behavioural risk factors, fiscal sustainability, healthcare expenditure growth, preventable diseases, risk-proportionate regulation, sugar-sweetened beverage taxation, tobacco harm reduction

## Abstract

**Introduction:**

Rising healthcare expenditure poses a major fiscal challenge for European governments, driven in part by preventable diseases associated with tobacco use, alcohol consumption, and sugar-sweetened beverages (SSBs). Although regulatory interventions are widely used to address these risk factors, most evaluations focus on overall policy stringency and implicitly assume homogeneity within product categories. This overlooks the potential importance of risk-proportional regulatory designs that differentiate products according to their relative health risks.

**Methods:**

This study investigates whether differences in the design of regulatory frameworks for tobacco, alcohol, and SSBs are associated with variations in healthcare spending growth across European countries. Specifically, it compares conventional measures of regulatory restrictiveness with a risk-proportional approach that calibrates regulatory burdens according to the relative risks of products within each category. The relationship between regulatory design, healthcare expenditure growth, and population health outcomes is examined using cross-country statistical analysis.

**Results:**

The analysis indicates that risk-proportional regulatory frameworks are more strongly associated with lower healthcare spending growth and improved population health outcomes than uniform measures of policy stringency. Regulatory systems that incentivize substitution toward lower-risk alternatives are associated with a more favourable overall risk profile of consumption.

**Discussion:**

These findings suggest that the design of health regulations, rather than their overall restrictiveness alone, may play an important role in promoting fiscal sustainability. By encouraging harm reduction within product categories, risk-proportional regulation may contribute to reducing the long-term burden of preventable diseases and the associated pressure on national healthcare budgets.

## 1 Introduction

In the wake of global health crises and shifting geopolitical tensions, national health systems face persistent pressure (1) from rising expenditures and fiscal sustainability concerns, leaving little room for secular challenges like aging populations and healthier lifestyles, shifting focus toward the resolution of immediate budgetary shocks (2–5). Yet, a substantial and addressable portion of this pressure is linked to preventable disease associated with behavioural risk factors, most prominently the consumption of alcohol, tobacco, and sugar-sweetened beverages (SSBs) (6–9). With tobacco responsible for more than 7 million deaths (10) each year, alcohol for about 2.6 million deaths (2019) (11), and dietary risks (including SSB intake) (12) for about 7.94 million deaths, prevention policy is central not only to population health, but also to healthcare cost management (13).

This paper asks whether risk-proportionate regulation of harmful goods is associated with better outcomes across Europe—specifically, more moderate growth in per-capita healthcare spending and improved health indicators. As explored in previous work (14), the efficacy of regulation depends on its ability to provide clear signals to the market. The hypothesis is that when policy burdens within a category align with relative risks, a risk-proportionate regulatory design correlates with a market shift toward lower-risk alternatives (15, 16). By incentivizing producers to pivot toward less harmful innovations and guiding consumers toward safer alternatives, this approach may reduce the downstream disease burden and associated fiscal pressure more effectively than uniform restrictions (14, 17).

Governments typically address these risk factors through policy bundles that typically include taxation, marketing restrictions, product regulation, and constraints on availability (18–20). Comparative assessments of these policies often summarize national approaches with overall implementation scores that emphasize aggregate restrictiveness rather than the internal structure of regulation (21, 22). Composite indices are thus useful for benchmarking, but they often assume that a single “stringency” dimension is sufficient to characterize the effectiveness of a national policy approach (23). This is plausible only if products within a category are similar in risk. In practice, markets for tobacco, alcohol, and SSBs include variants with meaningfully different risk profiles. The relevant margins for health improvement are often found within-category: combustible vs. non-combustible nicotine products; varying ethanol content in alcohol; and sugar concentration under tiered SSB taxes (24). Because existing cross-country scorecards fail to capture whether a policy mix is “flat” or “calibrated” to these internal risk gradients, they often obscure the primary mechanism of modern prevention: the scope for substitution (25). To address this measurement gap, we introduce SCORE (Standardized Comparative Risk Evaluation) to quantify risk-proportionate differentiation and relate it directly to spending and health outcomes.

For cost management, this distinction matters. A “uniform” approach^1^ raises burdens broadly within a category, regardless of variation in risk. Conversely, a risk-proportionate approach calibrates fiscal and non-fiscal burdens to within-category risk gradients, creating a structural incentive for substitution by making relative risks salient through price and regulatory signals (26). This shift directly moderates healthcare spending by transitioning the consumer base toward products with a significantly lower impact on public health budgets. The policy question is therefore not only whether regulation is “tough”, but whether it makes relative risks actionable for consumers.

Several governments have already incorporated risk-proportionate elements to support such substitution. Some calibrate taxes and non-tax restrictions to risk gradients, while others apply broad restrictions with little differentiation within product categories. Notably, this lack of consistency persists even at the national level, where regulatory strategies frequently differ across tobacco, alcohol, and SSBs. For example, the United Kingdom has historically maintained a distinct regulatory and fiscal environment for non-combustible nicotine products (27). This was designed to encourage substitution away from combustible cigarettes (28–30). The latest decision of a Generational Sales Ban seems to partially change this orientation, given the exclusion of some alternatives from the list of allowed products (31). While it is too early to make a data-based judgment on this newly approved measure, its introduction highlights the need for a clear metric, like SCORE, to track how policy reforms move a country along the risk-proportionate design scale.

By capturing within-category differentiation rather than aggregate restrictiveness, SCORE allows for the quantification of these policy shifts comparably. We build domain scores for alcohol, tobacco, and SSBs and aggregate them into a country-level index, where higher values indicate stronger alignment of policy burdens with risk gradients. We then examine whether a higher SCORE is associated with (i) more moderate growth in per-capita healthcare spending (PPS per inhabitant) and (ii) more favourable trends in selected health indicators, particularly for tobacco and alcohol. The study proceeds by first normalizing heterogeneous regulatory data into the SCORE metric and subsequently applying regression analysis to evaluate the association between these design choices and health spending data across European countries between 2014 and 2022.

The results suggest that risk-proportionate differentiation may offer a targeted policy instrument for improving fiscal and health outcomes without necessarily requiring a uniform increase in category.

The remainder of the paper is organized as follows. Section 2 details the data and the methodological framework used to construct the SCORE index. Section 3 presents the empirical findings, and Section 4 discusses their policy implications. Finally, Section 5 addresses the study's limitations and suggests priorities for future research, followed by concluding remarks in Section 6.

## 2 Data

To estimate the relation between regulatory differentiation and (i) risk-attributable health outcomes and (ii) per-capita healthcare spending growth, we conducted a cross-country analysis of 30 European countries. Inclusion in the sample was contingent upon the availability of policy information required to construct the Standardized Comparative Risk Evaluation (SCORE) index and the presence of relevant outcome indicators over the specified timeframe.^2^ Due to varying data availability across these indicators (e.g., 2011–2021 for risk-attributable DALYs/YLDs and 2014–2022, or the latest available year, for per-capita healthcare spending), the effective sample size varies across models.

Our primary focus is the change in per-capita health expenditure measured in Purchasing Power Standards (PPS) per inhabitant, calculated between 2014 and 2022 (or the latest available year) (32). This variable is selected for its close connection to the policy-relevant spending pressures facing health systems.

To capture potential health outcomes associated with differentiated regulation, we use standard burden-of-disease measures, specifically DALYs and YLDs, constructed either for overall behavioural risks or for domain-aligned risk factors related to tobacco, alcohol, and sugar-related consumption (33). In addition, we use complementary indicators, such as life expectancy and selected prevalence measures, as robustness checks. These outcomes help assess whether SCORE is associated not only with spending dynamics but also with measurable changes in population health.

**Figure 1:**
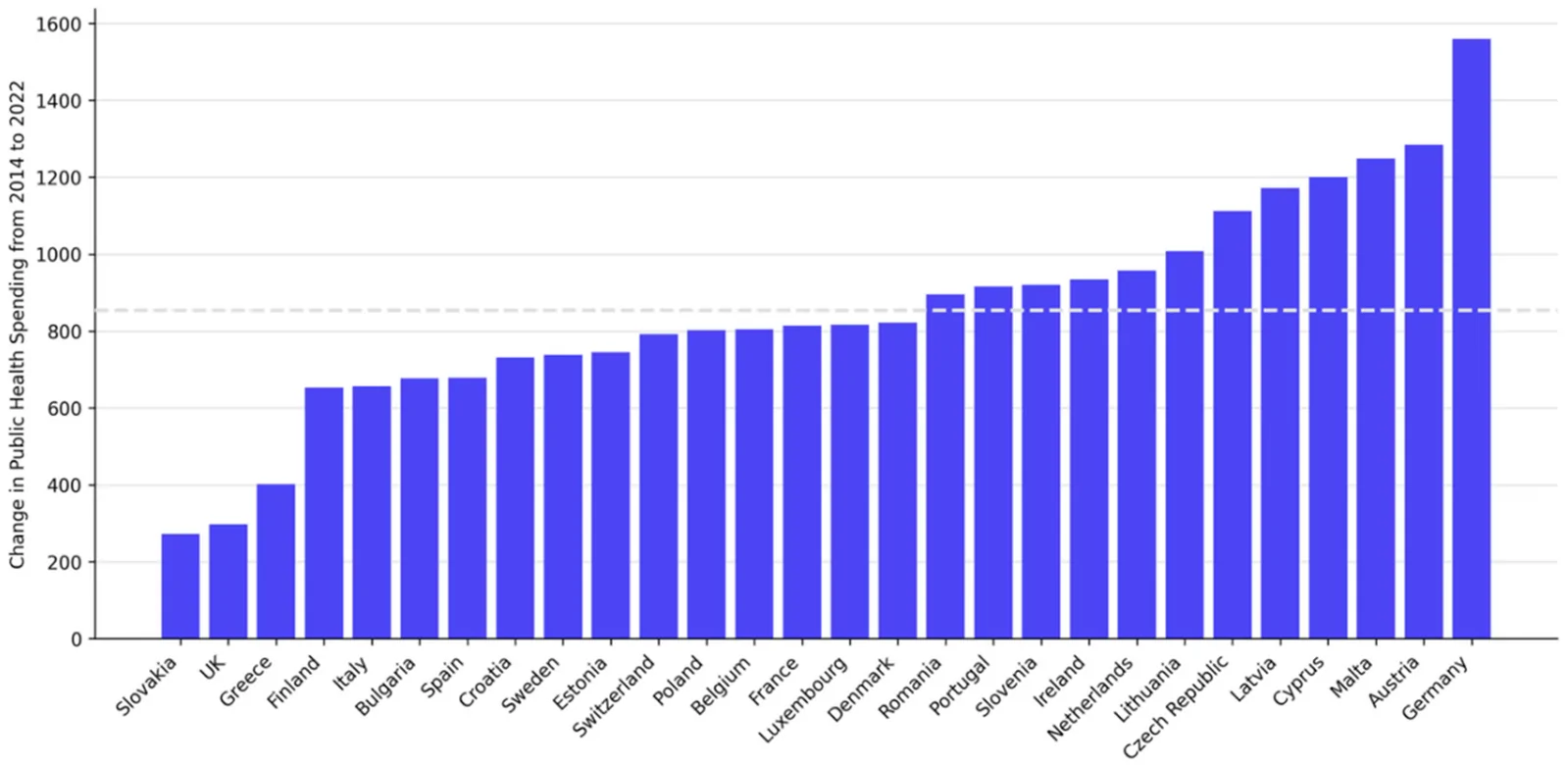
Cross-country dispersion in healthcare spending growth from 2014 to 2022, PPS per capita. This figure shows the change in per-capita healthcare expenditure (PPS per inhabitant) from 2014 to 2022 (or latest available year) for each country in the sample. Countries are ordered by the magnitude of the spending change to highlight cross-country dispersion. The dashed line shows the cross-country mean.

Figure 1 plots the change in per-capita healthcare expenditure (PPS per inhabitant) between 2014 and 2022 across the sample, revealing a highly dispersed distribution. The distribution is highly dispersed, with the lowest-growth countries exhibiting increases of only a few hundred PPS per inhabitant, while the upper tail, led by Germany, reaches roughly 1,500–1,600 PPS per inhabitant. Although a dense cluster of countries appears in the middle of the distribution (between 600 and 1,000 PPS), the overall data confirms substantial cross-country heterogeneity. In other words, this variation highlights that healthcare spending growth differs markedly across European countries, likely driven by distinct national policy designs. We leverage this variance to investigate whether risk-proportionate regulation, measured through SCORE, provides an effective lever for moderating these expenditure trajectories.

To evaluate the relationship between regulatory design and these divergent spending trajectories, we construct SCORE, a composite index of within-category differentiation in policy burdens across three domains: tobacco, alcohol, and SSBs. The framework rests on the principle that because products within each domain carry varying risk profiles, an efficient policy environment imposes relative burdens that are proportionate to risk: taxing and regulating higher-risk products more strictly while keeping lower-risk substitutes comparatively less burdened

For each domain, we assign points for policy features that differentially tax or regulate product variants in alignment with risk gradients (e.g., differentiated excise tax structures, product-specific restrictions). These domain scores are aggregated into an overall index using weights (40% tobacco; 30% alcohol; 30% SSBs) and scaled from 0 to 100. Consequently, a higher SCORE indicates a more risk-proportionate differentiated policy environment, rather than the overall stringency of the regulation. Detailed scoring rubric and country-by-country components are reported in Supplementary Appendix A.

The allocation of domain weights reflects the relative burden of disease attributable to each category in the European context. Tobacco receives the highest weight (40%) as the leading preventable cause of mortality in Europe, responsible for approximately 700,000 deaths annually (WHO, (10)); this is broadly consistent with estimates placing tobacco as the largest single contributor to healthcare costs among the three domains. Alcohol and sugar-sweetened beverages are each assigned 30%, reflecting their comparably documented health and economic burden (WHO, (11); IHME, (33)). As a robustness check, Supplementary Appendix Table A4 reports results under equal weights (33.3% each); the Spearman rank correlation between the two schemes is 0.97 (*p* < 0.001), confirming that the main findings are not sensitive to the weighting choice.

Within each domain, individual indicators are scored relative to the maximum observed value in the sample. This approach facilitates cross-country comparison within the current dataset but introduces sample dependence: adding or removing countries might alter individual SCORE values, limiting direct comparability across different samples or time periods. As a sensitivity check, Supplementary Appendix Table A4 also reports results under z-score normalization of domain indicators prior to aggregation; the rank correlation with the original approach is 0.94 (*p* < 0.001), confirming that the normalization choice does not materially affect country rankings.

**Figure 2:**
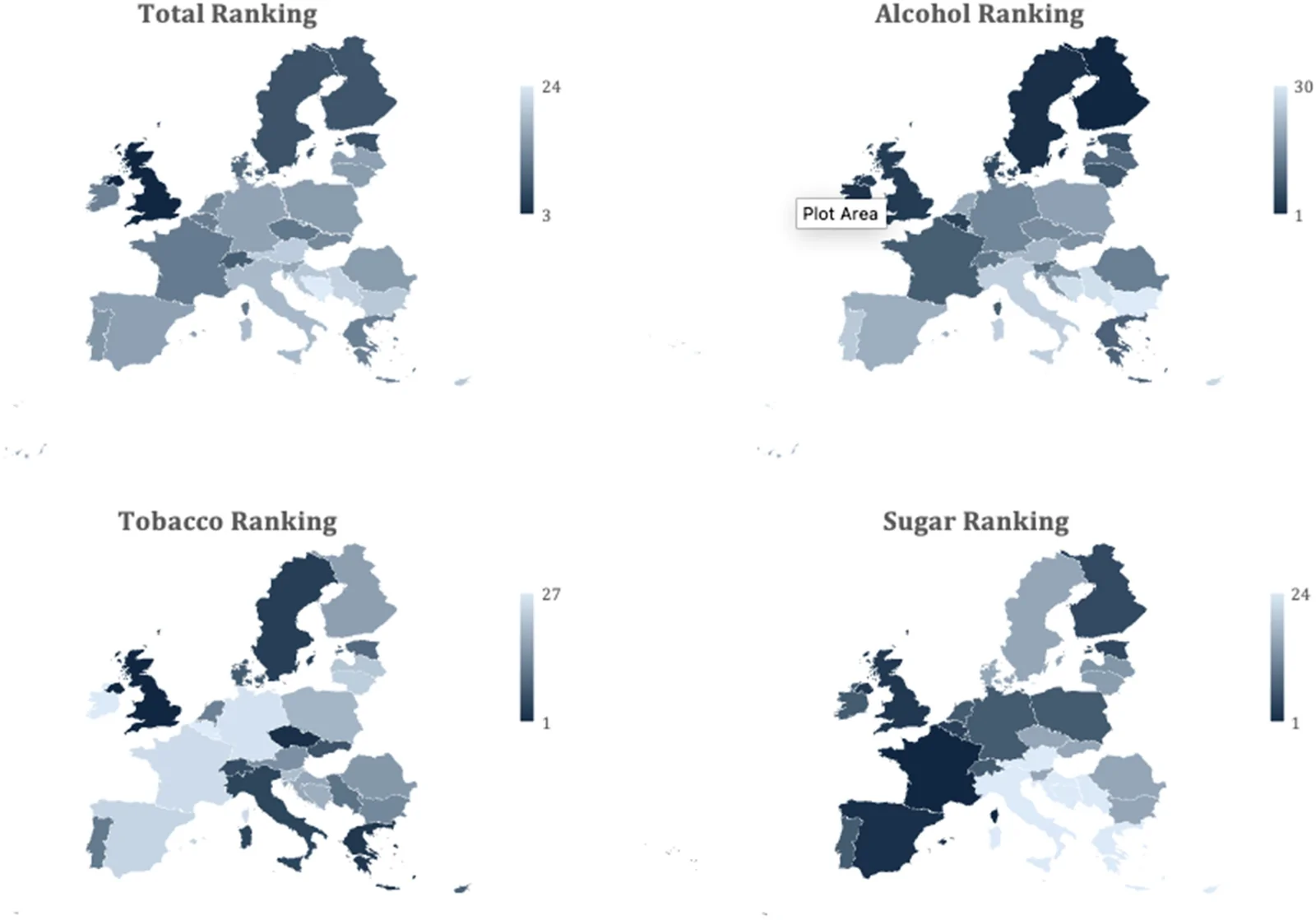
Ranking of countries by variation in risk-proportionate policy differentiation (SCORE). This figure maps the domain-specific SCORE index ranking for tobacco, alcohol, and sugar-sweetened beverages (SSBs), as well as the overall SCORE (weighted composite). Darker shading indicates greater within-category regulatory differentiation (i.e., more risk-proportionate design), not greater overall policy stringency. The underlying scoring rubric and country-level components are reported in Supplementary Appendix A.

Figure 2 summarizes cross-country variation in SCORE, revealing two primary patterns with significant implications for regulatory design. First, the substantial variance in scores across countries suggests that risk-proportionate policy design is an empirically distinct dimension of regulation. Second, domain scores are imperfectly correlated: countries that differentiate heavily in one domain do not necessarily do so in others. This underscores that SCORE captures deliberate, sector-specific policy choices, highlighting where the scope for risk-based substitution remains underutilized.

Table 1 further illustrates this within-country heterogeneity. While some countries, such as Germany, display relatively uniform differentiation across all three domains, others, for instance, Italy and Greece, exhibit pronounced imbalances. In these cases, high differentiation in one category (e.g., tobacco) is offset by minimal or absent differentiation in another (e.g., alcohol). Such non-uniformity is a critical diagnostic finding: an aggregate score can mask significant domain-specific gaps where policy reform could better align regulations with risk gradients. In the following sections, we leverage this variation to examine the association between policy differentiation, healthcare spending growth, and behavioral outcomes.

**Table 1:** Illustrative domain SCORE profiles (balanced vs. unbalanced differentiation).

Country	Tobacco SCORE	Alcohol SCORE	Sugar/SSB SCORE	Total SCORE
UK (balanced)	73.0	51.3	45.0	58.1
Germany (balanced)	19.5	17.2	25.0	20.5
Estonia (balanced)	54.5	26.3	30.0	38.7
Italy (unbalanced)	67.3	8.0	0.0	29.3
Greece (unbalanced)	69.7	22.2	0.0	34.5

Domain scores are on a 0–100 scale. Total SCORE is the composite. “Balanced” and “unbalanced” refer to the degree of within-country dispersion across domains, as illustrated by the examples in Table 1. The underlying scoring rubric and country-level components are reported in Supplementary Appendix A.

## 3 Results

This section examines the relation between differentiated, risk-proportionate regulation, captured by the SCORE index, and two primary outcomes: (i) the growth of per-capita healthcare spending and (ii) selected population health indicators linked to behavioural risks. The analysis proceeds in three steps. First, we provide a descriptive overview by reporting correlations between SCORE (overall and domain-specific) and our primary outcome measures. Second, we assess the consistency of these patterns through robustness checks using supplementary indicators, including life expectancy and prevalence-based measures disaggregated by gender, where data allow. Finally, we estimate multivariate regression models to measure the relationship between spending growth and SCORE, while controlling for a parsimonious set of country-level covariates.

### 3.1 Correlation analysis

We begin by examining the correlations between SCORE and our selected spending and health indicators. This descriptive approach provides an interpretable first-pass summary of whether countries with more risk-proportionate regulatory differentiation exhibit systematically different spending dynamics or health-burden trajectories.

**Figure 3:**
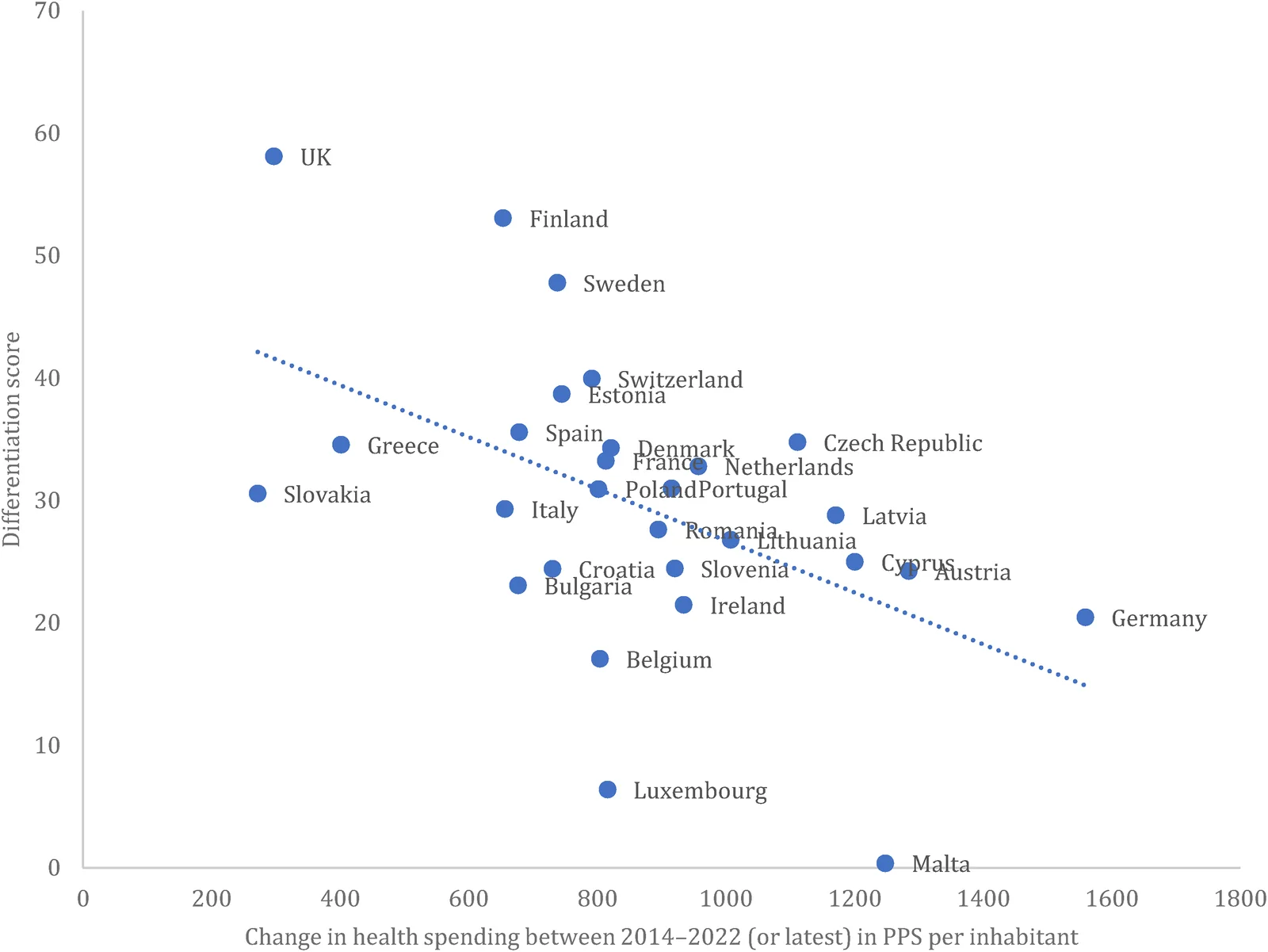
Correlation between SCORE and change in healthcare costs. This figure shows the cross-country association between SCORE (0–100; risk-proportionate within-category policy differentiation, not overall stringency) and the change in per-capita healthcare expenditure (PPS per inhabitant) over 2014–2022 (or latest available year). Each point represents one country; the line reports the fitted linear relation.

To anchor this analysis in a policy-relevant spending outcome, we first correlate the overall SCORE with the change in per-capita healthcare expenditure over 2014–2022. As shown in Figure 3, the relation is negative, with countries possessing higher SCORE cluster around a downward-sloping trend. In our sample, the correlation is approximately −0.51 (*p*-value = 0.01), suggesting that more differentiated regulatory environments are associated with more moderate increases in healthcare spending per inhabitant over the period.

We then assess whether SCORE is also associated with population health measures linked to alcohol, tobacco, and sugar consumption. We focus on changes in selected burden-of-disease metrics (DALYs/YLDs, where available) and on auxiliary outcomes such as life expectancy and prevalence-based measures. Where relevant, we further disaggregate by gender to account for differences in baseline risk exposure and consumption patterns across men and women.

Table 2 summarizes correlations between SCORE and the selected health outcomes. The results are broadly consistent with our theoretical framework: higher differentiation is associated with favorable trends across several risk-related indicators, though the strength of these associations varies by domain and metric. In particular, alcohol-specific differentiation exhibits the strongest associations with indicators of risky drinking behavior. Tobacco differentiation shows directionally similar, albeit moderate, correlations across morbidity measures. In contrast, sugar-related correlations tend to be the lowest, likely reflecting the relatively more recent adoption of SSB-targeted policies in many countries and the expected lags between policy implementation and downstream health outcomes.

**Table 2:** Correlations between SCORE and healthcare spending and health outcomes.

SCORE	Health (spending) indicator	Correlation (90%-confidence interval)
Total	Change in healthcare spending 2014–2022 (or latest) in PPS per inhabitant	−0.51 (0.32, 0.98)
Tobacco	Change in the share of behavioural risk related YLDs, 2011–2021	−0.28 (−0.58, 0.03)
Alcohol	Change in the proportion of total YLDs attributable to alcohol, 2011–2021	−0.63 (−0.81, −0.35)
Alcohol	10-year difference in heavy episodic monthly drinking, 2011–2021	−0.52 (−0.74, −0.2)
Sugar	Change in the share of SSB-related deaths from non-communicable diseases among women, 2011–2021	−0.35 (−0.63, 0.01)
Sugar	Change in the share of SSB-related DALYs among men, 2011–2021	−0.32 (−0.61, 0.04)

This table shows bivariate (Pearson) correlations between SCORE (overall and/or domain-specific) and selected health indicators. Correlations are computed across countries; each row reports the correlation for the outcome listed in column (2). Higher SCORE indicates greater within-category regulatory differentiation (risk-proportionate design), not overall policy stringency.

To assess whether these descriptive relations are sensitive to the choice of outcomes, we perform additional checks using (i) aggregate measures of population health and (ii) risk-factor-aligned prevalence measures.

First, we use life expectancy at birth as a summary indicator. In our sample, life expectancy is positively correlated with the overall SCORE, suggesting that countries with higher regulatory risk-proportionate differentiation across alcohol, tobacco, and SSB also have higher baseline levels of population health. This finding motivates the inclusion of baseline health controls in the multivariate analysis to account for potential confounding.

Second, we use prevalence-based measures that directly reflect behavioural risks. Regarding tobacco, the correlation between tobacco-specific differentiation and changes in male smoking prevalence is negative, consistent with the hypothesis that higher differentiation is associated with lower smoking rates. For alcohol, the association is stronger. The alcohol SCORE is negatively correlated with shifts in heavy episodic drinking (Table 3), suggesting that differentiated alcohol policies coincide with more favourable trends in risky consumption behaviour.

**Table 3:** Correlation between SCORE and health outcomes.

SCORE	Health Indicators	Correlation (95%-confidence interval)
Total	Change in the share of behavioural risk-related YLLs (neoplasms) among women, 2011–2021	−0.40 (−0.67, −0.05)
Total	Life expectancy at birth	0.37 (0.10, 0.64)
Tobacco	Change in male smoking prevalence, 2010–2022	−0.24 (−0.55, 0.13)
Alcohol	Change in weekly heavy episodic drinking, 2014–2019	−0.63 (−0.81, −0.35)
Sugar	Life expectancy at birth	0.37 (0.07, 0.78)

This table shows bivariate correlations between SCORE (overall and domain-specific) and selected health-burden indicators over the windows indicated in the row labels. Higher SCORE indicates greater risk-proportionate within-category differentiation, not greater overall policy strictness.

### 3.2 Multivariate analysis of healthcare spending growth

We next quantify the relationship between SCORE and healthcare spending growth in a multivariate framework. The dependent variable is the change in per-capita health expenditure (PPS per inhabitant) from 2014 to 2022, with the aggregate SCORE serving as the independent variable. Given the observational nature of this cross-country analysis, we acknowledge that differences in economic capacity, demographics, and baseline health-system performance likely correlate with both policy design and spending trajectories. To mitigate potential omitted variable bias, we estimate a parsimonious model that controls for these specific channels.

We include GDP per capita and the employment ratio to capture income levels and labour market participation that may affect both healthcare demand and the administrative capacity to implement differentiated policies. We include life expectancy at birth and infant mortality as proxies for baseline health outcomes and system performance. These variables may influence both the political demand for preventive regulation and the level and growth rate of healthcare spending. We further include internet use and the urban population share to capture modernization and the structure of service delivery, which can affect both health behaviours and the capacity for policy monitoring and enforcement. Table 4 provides definitions for all variables used in the regression analysis.

**Table 4:** Regression variables.

Variable	Description
Change in healthcare spending 2014–2022 (or latest) in PPS per inhabitant	Change in health expenditure per capita in PPS from 2014 to 2022.
Regulatory score	Reflects perceptions of the extent to which a country's citizens are able to participate in selecting their government, as well as freedom of expression, freedom of association, and a free media.
GDP per capita (current USD)	Per capita gross domestic product (GDP) expressed in current international dollars converted by purchasing power parity (PPP) conversion factor.
Employment to population ratio, 15+, total (%)	Employment to population ratio is the proportion of a country's population that is employed. Ages 15 and older are generally considered the working-age population.
Government expenditure on education, total (% of GDP)	General government expenditure on education (current, capital, and transfers), expressed as a percentage of GDP.
Mortality rate, infant (per 1,000 live births)	Infant mortality rate is the number of infants dying before reaching one year of age, per 1,000 live births in a given year.
Urban population (% of total population)	Urban population refers to people living in urban areas as defined by national statistical offices. The data are collected by United Nations Population Division.
Individuals using the Internet (% of population)	The percentage of people who use the Internet from any location and for any purpose, irrespective of the device and network technology used.
Consumer price index (2010 = 100)	Consumer price index reflects changes in the cost to the average consumer of acquiring a basket of goods and services that may be fixed or changed at specified intervals, such as yearly.
Life expectancy at birth	Life expectancy at birth indicates the number of years a newborn infant would live if prevailing patterns of mortality at the time of its birth were to stay the same throughout its life.

This table shows variable definitions and measurement windows.

We begin with a baseline specification relating spending changes to SCORE, subsequently adding controls in a stepwise manner. Across all specifications (Table 5), the SCORE coefficient is negative and statistically significant at 1% level. This result indicates that countries with higher regulatory differentiation experienced smaller increases in healthcare spending per inhabitant during the period. Specifically, a 10-point higher SCORE is associated with an avoided increase in healthcare spending of approximately EUR 64.4 to EUR 67.6 per capita annually.

**Table 5:** SCORE and growth in per-capita healthcare spending.

Healthcare spending	A	B	C	D
SCORE	−6.44***	−6.59***	−6.76***	−6.60***
	(1.33)	(1.34)	(1.40)	(1.38)
Employment ratio	20.96***	20.32***	17.24*	20.14**
	(7.57)	(7.58)	(9.59)	(7.92)
Mortality rate	−101.72**	−144.80**	−155.16**	−113.52**
	(42.42)	(44.18)	(44.91)	(46.58)
CPI 2010		5.97	7.84	6.43
		(5.75)	(6.79)	(7.13)
GDP per capita			0.0014	
			(0.0025)	
Life expectancy				2.18
				(19.15)
Intercept	565.26	−31.50	−111.48	−251.49
	(479.98)	(748.08)	(774.90)	(2,080.42)
R^2^	0.57	0.59	0.59	0.59
Adjusted R^2^	0.51	0.52	0.49	0.49

The dependent variable is the change in per-capita healthcare expenditure (PPS per inhabitant) over 2014–2022 (or latest available year). Robust standard errors are reported in parentheses. *, **, *** denote statistical significance at the 10%, 5%, and 1% levels.

Regarding the controls, the employment ratio is positively associated with healthcare spending growth in most specifications, consistent with the hypothesis that higher employment is correlated with income, utilization, and/or the state's capacity to finance healthcare expansion. In contrast, infant mortality enters negatively in several specifications. This pattern is plausible in a cross-section because countries with higher infant mortality often exhibit both lower baseline spending levels and slower spending growth, reflecting persistent differences in income and health-system capacity.

The explanatory power of the models ranges from 0.57 to 0.59. The adjusted R² values are lower (0.49–0.51), suggesting that additional controls offer limited explanatory power beyond the primary predictors.

Table 6 includes further robustness checks. Other control variables, such as GDP per capita, consumer price index, life expectancy, urban population share, education expenditure, and internet use, do not display robust or consistent associations with healthcare spending growth. Their coefficients fluctuate in magnitude and statistical significance, suggesting that they do not independently drive differences in expenditure growth once regulatory quality, employment, and infant mortality are accounted for.

**Table 6:** Robustness checks: SCORE and healthcare spending growth.

Spending growth	A	B	C	D	E
SCORE	−6.88***	−6.96***	−6.83***	−6.86***	−6.98***
	(1.54)	(1.57)	(1.48)	(1.56)	(1.62)
Employment ratio	17.98	18.88	19.13	18.85	17.79
	(10.84)	(11.15)	(11.48)	(12.11)	(12.62)
Mortality rate	−120.64**	−133.99**	−123.66**	−123.83**	−133.18**
	(50.28)	(56.32)	(51.32)	(52.74)	(58.02)
CPI 2010	5.40	5.25	7.73	7.51	5.895
	(7.67)	(7.82)	(7.12)	(7.62)	(8.62)
GDP per capita			0.001	0.0004	0.0009
			(0.003)	(0.004)	(0.004)
Life expectancy	−6.57	−11.04			−12.42
	(25.12)	(26.76)			(28.31)
Urban population	2.00	3.44	2.22	2.25	3.18
	(4.01)	(4.81)	(4.40)	(4.53)	(5.10)
Education expenditure		−39.07	−22.86	−25.92	−30.29
		(68.69)	(72.28)	(80.00)	(82.47)
Internet use	3.47	4.68		1.49	3.06
	(12.41)	(12.82)		(14.59)	(15.35)
Intercept	278.65	634.86	−193.33	−259.68	817.12
	(2,350.60)	(2,474.16)	(845.41)	(1,082.89)	(2,692.69)
R^2^	0.59	0.61	0.60	0.601	0.606
Adjusted R^2^	0.45	0.43	0.45	0.424	0.397

The dependent variable is the change in per-capita healthcare expenditure (PPS per inhabitant) over 2014–2022 (or latest available year). Robust standard errors are reported in parentheses. *, **, *** denote statistical significance at the 10%, 5%, and 1% levels.

The regression estimates indicate that countries with greater risk-proportionate differentiation in harmful-goods regulation were associated with more moderate increases in per-capita healthcare spending over the analyzed period. There are at least three interpretations consistent with the observed pattern.

#### 3.2.1 Substitution and risk composition

**Figure 4:**
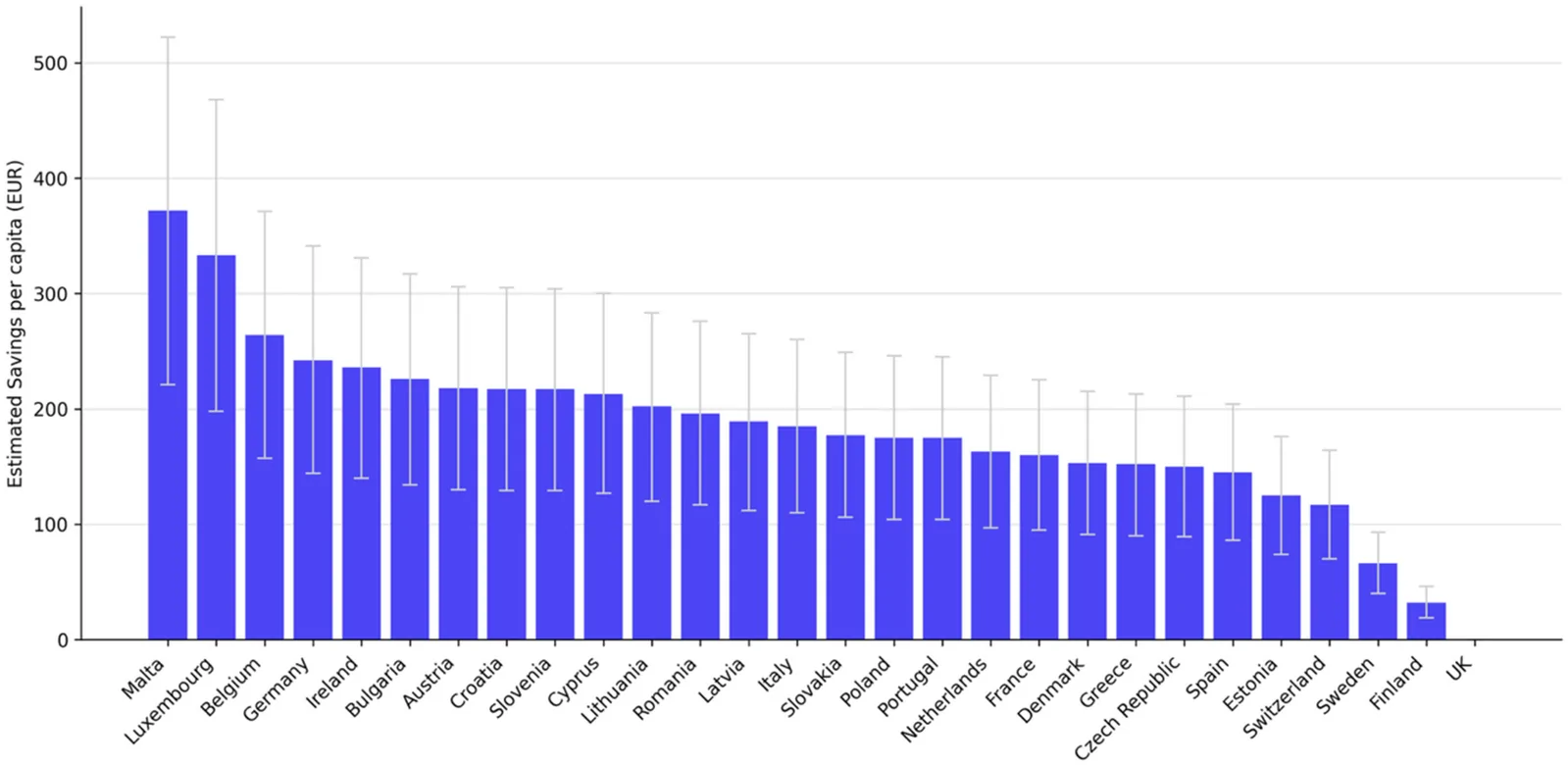
Per capita healthcare spending savings from higher regulatory differentiation. This figure shows implied per-capita healthcare spending savings (PPS per inhabitant) from increasing regulatory differentiation to the UK benchmark. For each country, savings are computed given the SCORE coefficient estimate of −6.44 from Table 5. Countries are ordered by magnitude of estimated savings per capita; the UK equals 0 by construction. Whiskers report 95%-confidence intervals.

**Figure 5:**
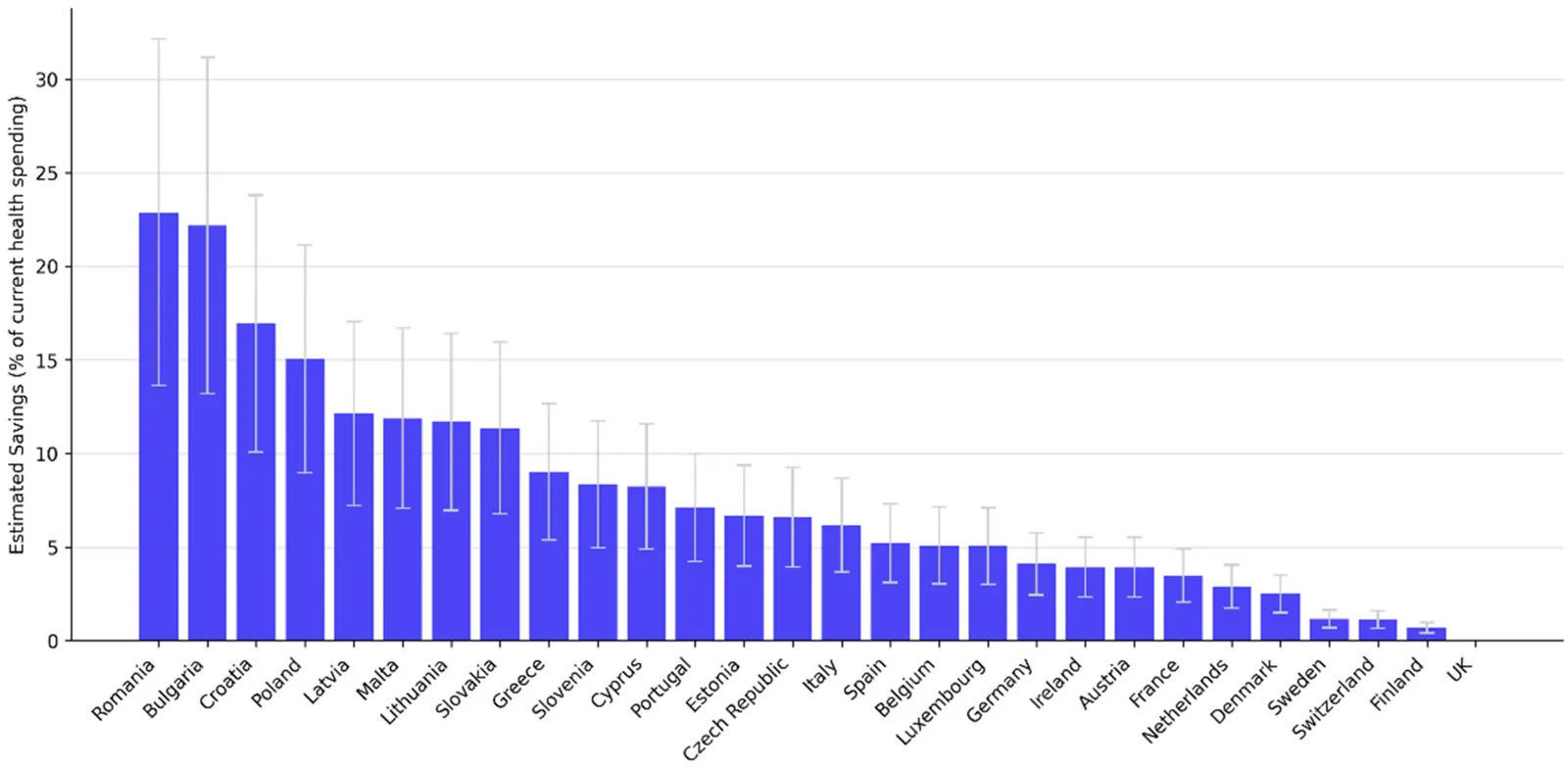
Healthcare spending savings from higher regulatory differentiation relative to current spending. This figure shows implied savings expressed as a share of current healthcare spending from increasing regulatory differentiation to the UK benchmark. For each country, savings are computed given the SCORE coefficient estimate of −6.44 from Table 5. Countries are ordered by implied savings; the UK equals 0 by construction. Whiskers report 95%-confidence intervals.

If differentiated policies shift consumption towards lower-risk variants within a category (e.g., within tobacco or alcohol), downstream morbidity and healthcare utilization may evolve more favourably, even in the absence of a significant or immediate reduction in total consumption volume. This mechanism is consistent with the conceptual motivation for SCORE as a measure of within-category differentiation rather than overall restrictiveness. Figures 4, 5 translate the SCORE-spending gradient into an economically interpretable object by mapping the model-implied (association-based) reduction in annual per-capita healthcare spending growth associated with closing each country's SCORE gap to the UK benchmark.

Using the SCORE coefficient estimate of −6.44 from Table 5, we compute for each country the implied reduction in annual per-capita healthcare spending growth (PPS per inhabitant) associated with reaching the UK benchmark (UK = 0 by construction); whiskers report 95%-confidence intervals around this estimate. Figure 4 shows that the implied savings are large and highly dispersed: per-capita savings range from approximately EUR 30 for Finland to roughly EUR 300–EUR 370 PPS per person per year in the upper tail (e.g., Malta and Luxembourg), with a broad middle group around EUR 150–EUR 230 PPS per person per year. These figures represent descriptive counterfactual scales derived from the linear gradient, rather than definitive projections of realized budgetary cuts.

Figure 5 expresses the same implied savings as a percentage of current healthcare spending. While Figure 4 emphasizes absolute PPS amounts, Figure 5 shows that countries with lower baseline spending can still realize substantial proportional savings even when their per-capita totals are not among the highest. The upper tail of the distribution reaches roughly 20% (Romania and Bulgaria), while many countries lie in the 5%–10% range; the UK again sits at zero by construction. As in Figure 4, implied savings are computed using the SCORE coefficient estimate of −6.44 from Table 5 and whiskers report 95%-confidence intervals.

To express these implied per-capita effects at the national level, we convert the benchmark-closing savings in Figure 5 into avoided healthcare expenditure by multiplying each country's implied savings share by its 2023 total healthcare expenditure. This yields sizeable but heterogeneous fiscal magnitudes (Supplementary Table A3). In absolute terms, avoided costs are largest in countries with high baseline spending: Germany (EUR 20,154.08 million), France (EUR 11,314.86 million), and Italy (EUR 11,089.76 million). At the lower end, Finland could avoid EUR 197.14 million annually (0.69% of 2023 healthcare spending), while the UK is zero by construction. At the same time, Figure 5 highlights that some countries with smaller absolute totals can nevertheless represent very large proportional savings: For Romania and Bulgaria, savings exceed 20% of current healthcare spending, and Croatia also implies a large savings share despite a comparatively smaller euro amount (Supplementary Table A3). In aggregate across the study area, the implied avoided healthcare expenditure amounts to EUR 85.99 billion. Consistent with the correlation patterns above, the largest share of this aggregate savings potential is concentrated in tobacco- and alcohol-related differentiation (tobacco: EUR 40.52 billion; alcohol: EUR 19.92 billion; see Supplementary Table A3).

While the preceding analysis illustrates the potential gains from closing the differentiation gap relative to a benchmark, the model can also be used to track the prospective impact of policy reversals. For example, applying a stylized scenario to the UK Generational Sales Ban (GSB) suggests that the UK tobacco sub-score would drop to one of the lowest in the sample. This would lower the total UK SCORE from 56.4 to 24.9 (*Δ*SCORE = −31.5). Mapping this change through our estimated gradient implies an illustrative increase in annual per-capita healthcare spending growth of approximately EUR 203–213 (PPS) per inhabitant (i.e., 3.15×EUR 64.4 to 3.15×EUR 67.6). This calculation is an association-based mapping intended to convey scale, not a causal forecast of the GSB's effects.

Interpreted through the substitution logic motivating SCORE, greater within-category differentiation can tilt relative prices and regulatory constraints across product variants, inducing substitution away from higher-risk variants and toward lower-risk alternatives. Such substitution may improve the composition of consumption, particularly in tobacco and alcohol, even when total consumption volumes adjust only gradually. In turn, downstream morbidity and healthcare utilization pressures may evolve more favourably, translating into slower growth in healthcare spending per inhabitant. Where the implied savings reach the magnitudes shown in Figures 4, 5, they are large enough to matter for state budgets, creating additional fiscal space and easing medium-term consolidation pressures. From a policy perspective, Figures 4, 5 therefore illustrate a potentially material potential in national healthcare savings associated with closing the differentiation gap, with the largest fiscal relevance where current policies remain furthest from the benchmark (i.e., where the SCORE gap is greatest). This “scope for improvement” suggests that introducing more differentiated instruments can facilitate a welfare-improving reallocation of consumption toward lower-risk variants without necessarily requiring a surge in overall regulatory stringency.

#### 3.2.2 Policy capacity and implementation quality

Risk-proportionate differentiation may serve as a proxy for broader state capacity. Countries able to design and administer nuanced tax schedules and product-specific regulations are more likely to implement broader health-system governance reforms that moderate spending growth. In this interpretation, SCORE functions partly as an index of administrative sophistication rather than a direct causal lever.

#### 3.2.3 Baseline health and institutional differences

Countries differ structurally in baseline health burden and in health-system cost growth, factors that may co-determine the political demand for risk-based prevention policies. The inclusion of baseline health proxies (life expectancy, infant mortality) reduces, but cannot fully eliminate, this concern. The stability of the SCORE coefficient across specifications suggests that differentiation captures spending growth above and beyond these coarse proxies, but the cross-sectional design does not permit a definitive causal claim.

## 4 Policy implications

This section summarizes the policy interpretation of the empirical patterns documented in Section 3. Our central finding is that countries with comparatively low within-category differentiation may be overlooking a critical opportunity to moderate healthcare spending growth by failing to align regulatory burdens with objective product risk. Even among countries ranking closer to the high-differentiation benchmark, where risk proportionality has been internalized to a greater extent, domain-specific SCORE rankings reveal significant internal inconsistencies. These data suggest that differentiation is often applied unevenly and could be implemented more systematically across both policy instruments and product categories.

The UK example is instructive from a policy perspective. Over the period covered by our data, the UK represents a high-differentiation benchmark, reflecting a long-standing emphasis on within-category risk gradients in tobacco control and harm reduction. The recent adoption of a GSB signals a structural pivot toward a “flat” regulatory model for future cohorts. Our stylized scoring suggests that such a transition toward non-differentiated, aggregate measures creates a “differentiation deficit” that effectively neutralizes the substitution channel. This shift corresponds to an illustrative increase in annual per-capita healthcare spending growth of approximately EUR 203–213 (PPS) per inhabitant. While not a causal forecast, this valuation highlights that abandoning risk-proportionate signals is not fiscally neutral. Rather, it risks eroding the long-term fiscal dividends previously secured through different strategies and sends mixed signals to the market. This “differentiation deficit” is not uniform across Europe, and the documented variance reveals significant unexploited policy potential. Some countries, and some domains within countries, already implement comparatively risk-proportionate differentiation. As such, closing the gap is not a theoretical exercise but a pragmatic lever for reform. This approach emphasizes recalibration in line with risk, rather than blanket increases in category-wide restrictiveness, making it a potentially more “consumer-friendly” path to expenditure containment. The diagnostic utility of SCORE is most evident when examining internal policy inconsistencies. Supplementary Table A2 illustrates how an overall proximity to the benchmark can mask distinct policy gaps. Czechia is ranked close to the frontier overall, driven by very strong differentiation in tobacco, but it is notably weaker in alcohol and SSB. This suggests that future policy gains could be realized by extending the risk-proportionate logic more consistently across all categories. France presents a different profile: it performs strongly in alcohol differentiation but lags in tobacco and sugar. In this case, a systematic approach would prioritize closing differentiation gaps in tobacco and sugar-related instruments rather than further tightening the already-differentiated alcohol policy.

The objective of such reforms is to ensure that lower-risk substitutes are not inadvertently penalized by regulations designed for high-risk products, thereby facilitating the substitution necessary to contain healthcare expenditures while maintaining robust safeguards against the initiation of these risky behaviors. At the EU level, this benchmark-closing exercise reflects massive fiscal stakes. Summing the implied avoided costs across all member states yields EUR 85.99 billion in potential avoided healthcare expenditure. This underscores that risk-proportionate differentiation is a critical priority for long-term fiscal sustainability at both the national and European scales.

## 5 Limitations

While this research provides a novel framework for evaluating regulatory design, several limitations warrant consideration. First and foremost, the analysis is strictly cross-sectional and therefore documents statistical associations rather than causal effects. Despite conditioning on a parsimonious set of country characteristics, unobserved institutional, behavioural, and health-system factors may jointly shape both regulatory design (and hence SCORE) and spending dynamics, implying potential omitted-variable bias. Reverse causality cannot be excluded either: countries with stronger institutions or more developed health systems may simultaneously exhibit higher SCORE values and distinct expenditure trajectories. Second, measurement limitations also remain. SCORE is constructed from policy features observed at a point in time and cannot fully capture enforcement intensity, subnational variation, or the impact of very recent policy changes. Furthermore, the outcome indicators differ in their availability windows across measures, and policy impacts are likely subject to heterogeneous lags, particularly in the SSB domain, where major instruments are relatively recent. As such, the long-term fiscal and behavioral effects of risk-proportionate SSB designs require further empirical validation as more longitudinal data becomes available.

No multi-domain index capturing risk-proportionate differentiation currently exists, precluding systematic external validation of SCORE against established composite regulatory or public health indices. As a partial check on face validity, tobacco domain scores correlate positively with country rankings from the Tobacco Control Scale 2021 [Joossens et al. (21)], the most widely used European tobacco policy index; however, unlike SCORE, the TCS measures overall regulatory restrictiveness rather than within-category risk-proportionate differentiation, so the comparison is imperfect. To the extent that SCORE is measured with error, reflecting unobserved enforcement intensity or subnational variation. standard errors-in-variables theory implies that the estimated coefficients are biased toward zero, meaning the reported associations likely understate the true relationship between risk-proportionate regulation and health expenditure outcomes. Sensitivity analyses presented in Supplementary Appendix Table A4 confirm that country rankings are robust to alternative weighting schemes (equal weights) and normalisation approaches (z-score).

SCORE combines continuous, ordinal, and binary indicators within a single additive framework, consistent with established composite index practice as employed by the Human Development Index (UNDP), the Tobacco Control Scale [Joossens et al. (21),], and the Nanny State Index (Snowdon, (23)). A methodological implication is that continuous components may contribute greater variance to the index than binary components, potentially affecting the relative influence of individual indicators on the aggregate score. Domain-level normalisation to a 0–100 scale prior to aggregation partially mitigates this asymmetry, but residual variance heterogeneity cannot be fully excluded. Beyond these statistical properties, this heterogeneity reflects the intrinsic nature of the evaluated policies. Regulatory and fiscal measures are, by definition, difficult to place on a common scale: their implementation and enforcement intensity vary substantially across countries, and the three domains capture fundamentally different behaviors and mechanisms. Aggregating these into a single composite index requires simplifying assumptions that cannot be fully avoided in cross-country comparative work of this kind.

It is also important to contextualize the use of the United Kingdom as the primary benchmark for our “implied savings” calculations. Throughout the observed period (2014–2022), the UK represented the empirical frontier of risk-proportionate regulatory design in Europe. While recent legislative shifts signal a move toward a more aggregate and less differentiated model, the UK's performance during the sample years provides the most robust baseline for quantifying the fiscal benefits of differentiation. Consequently, the “gap-closing” exercises presented in this study should be interpreted as reaching a high-differentiation standard established during the period of analysis rather than a reflection of the UK's current or prospective policy trajectory.

Finally, the benchmark-based “implied savings” figures are illustrative counterfactual calculations. These estimates hold other country characteristics constant and do not incorporate general-equilibrium responses, implementation constraints, or political-economy frictions. Their purpose is to visually translate the estimated statistical gradient into a comparable scale rather than to provide country-specific forecasts. Addressing these gaps, specifically through longitudinal data to isolate causal mechanisms and micro-level consumption data to validate substitution patterns, remains a priority for future research.

## 6 Conclusion

This paper examines whether risk-proportionate regulatory design in tobacco, alcohol, and sugar-sweetened beverages is associated with more moderate growth in per-capita healthcare spending across Europe. By introducing SCORE to quantify within-category differentiation for 30 countries, we document substantial heterogeneity in policy design and identify a robust, statistically significant negative association between risk-proportionality and expenditure growth (PPS per inhabitant) over the 2014–2022 period.

These findings provide empirical validation for the hypothesis that policy design is a factor associated with fiscal sustainability outcomes. The observed patterns are consistent with a substitution-led mechanism. While the cross-sectional nature of this analysis necessitates an associational rather than a causal interpretation, the magnitude and stability of the results are robust and policy-relevant. Our analysis suggests that the “differentiation gap” represents a critical opportunity for European health systems. Improving within-category differentiation is far from a theoretical preference; it is a pragmatic, high-impact reform lever for governments facing persistent budgetary pressures. SCORE provides the transparent, diagnostic benchmarking required to identify these unexploited policy gaps and track the fiscal implications of regulatory shifts.

## Data Availability

The raw data supporting the conclusions of this article will be made available by the authors, without undue reservation.

## Footnotes

^1^These indices include the Nanny State Index by European think tanks, the alcohol policies scale by the WHO, and the tobacco control scale by the European Union.

^2^Austria, Belgium, Bosnia & Herzegovina, Bulgaria, Croatia, Cyprus, Czechia, Denmark, Estonia, Finland, France, Germany, Greece, Ireland, Italy, Latvia, Lithuania, Luxembourg, Malta, Netherlands, Poland, Portugal, Romania, Serbia, Slovakia, Slovenia, Spain, Sweden, Switzerland, and the United Kingdom.
