# Supplementary material for: Differentiated regulation and health spending growth: European evidence from the new SCORE index
Source: Front Health Serv. 2026 Aug 12;6:1888179. doi: 10.3389/frhs.2026.1888179 (PMC13506421; doi:10.3389/frhs.2026.1888179)
Supplement: Supplementary file 1 [file Supplementaryfile1.docx]

## Appendix

A1. General scoring approach

For each domain, we define a set of regulatory dimensions in which countries may treat products differently within the same risk class. Countries receive points when differentiation exists along these dimensions (e.g., differentiated excise rates, differentiated advertising or sales restrictions, differentiated labelling requirements). Non-tax items generally receive fixed points when differentiation is present, while tax items are treated using a normalization procedure to ensure cross-country comparability. Specifically, for tax components we scale country scores relative to the maximum observed differentiation in the sample: the country exhibiting the highest tax differentiation receives the maximum points allocated to that tax component, and other countries receive points proportionally to their differentiation relative to the maximum. This normalization allows the index to preserve information on the degree of differentiation, rather than merely its presence, while keeping the domain score bounded between 0 and 100.

A2. Product-specific construction

**Alcohol (0–100).** The alcohol sub-index leverages differentiation across beverage types, beer, wine, and spirits, reflecting differences in alcohol content and associated risks. The tax component is operationalized through beverage-specific excise rates, scaled relative to the highest-tax country for each beverage type and then mapped into points (beer, wine, spirits with distinct maxima). Additional points are awarded when countries implement tax incentives for low- or no-alcohol beer and when key regulatory measures differentiate by product type (e.g., advertising restrictions and minimum age rules) and when drink-driving rules are stricter as captured by lower blood alcohol concentration limits.

**SSBs (0–100).** The sugar sub-index focuses on differentiation in fiscal and informational policies that may steer consumers towards lower-sugar choices. Countries receive the highest points when taxes on SSBs explicitly vary by sugar content (tiered designs), and fewer points when taxes are uniform and do not differentiate by sugar concentration. The index also awards points for the presence of front-of-pack nutrition labelling systems (e.g., Nutri-Score (34) or analogous traffic light schemes), restrictions on marketing to youth, and selected product environment measures such as restrictions on energy drinks and vending machines. Consistent with the scope defined in this first version of the index, we do not treat substitution towards low/no-calorie sweeteners as unambiguously beneficial and therefore do not explicitly score such substitution as a risk-reduction pathway within the sugar domain.

**Tobacco (0–100).** The tobacco sub-index captures differentiation between conventional cigarettes and novel tobacco products (notably heated tobacco products) across several regulatory areas, including taxation, packaging and labelling requirements, smoke-free environments, retail/sales restrictions, and sponsorship rules. Tax differentiation receives the dominant weight within this domain (maximum 60 points), with remaining points assigned for regulatory exemptions or differential treatment of HTPs relative to cigarettes. In addition, we incorporate an overall market stance component that assigns negative points to very restrictive approaches (e.g., bans) and positive points to risk-reduction–oriented approaches, reflecting the direction of policy towards non-combustible alternatives.

**Table A1.: Scoring Mechanism**

| **Regulatory Measures** | **Scoring (max. = 300)** | **Sources** | **Year** |
| --- | --- | --- | --- |
| **Tobacco (max. = 100)** |  |  |  |
| Taxation | Country with highest tax differentiation scores 60. The other countries’ scores are based on their tax differential as a percentage of the highest taxing country. | [Tax Foundation](https://taxfoundation.org/data/global-tax/eu-tax/);  [UK tobacco duty rates](https://www.gov.uk/government/publications/rates-and-allowances-excise-duty-tobacco-duty/excise-duty-tobacco-duty-rates);  [European Commission Tax Database](https://ec.europa.eu/taxation_customs/tedb/#/home) | 2023  2023  2024 |
| Packaging & labelling | Countries score if HTPs are subject to weaker requirements than cigarettes. This includes classifying devices as consumer products (+5), requiring smaller text health warnings than 65% (+2.5), or exempting HTPs from plain packaging rules (+2.5). | [Tobacco Control Laws](https://www.tobaccocontrollaws.org/legislation) | 2022 or latest |
| Smoke-free environments | Countries score if HTPs are exempt from smoke-free rules that apply to cigarettes in bars/pubs (+1), schools (+1), healthcare facilities (+1), public transport (+1), or workplaces/offices (+1). | [Tobacco Control Laws](https://www.tobaccocontrollaws.org/legislation) | 2022 or latest |
| Sale | Countries score if HTPs face fewer restrictions than cigarettes–for example, being sold in vending machines (+5), without limits in retail stores (+2.5), or with visible point-of-sale displays (+2.5). | [Tobacco Control Laws](https://www.tobaccocontrollaws.org/legislation) | 2022 or latest |
| Sponsorship | Countries score if HTPs are permitted to sponsor events/publicity (+2.5) or individuals (+2.5) where cigarettes cannot. | [Tobacco Control Laws](https://www.tobaccocontrollaws.org/legislation) | 2022 or latest |
| Market | Countries are scored based on their overall stance towards HTPs: very restrictive approaches (e.g., bans) = -10, risk-reduction–oriented approaches (e.g., classifying as risk-reduced) = +10, neutral approaches = 0. | [Tobacco Control Laws](https://www.tobaccocontrollaws.org/legislation) | 2022 or latest |
| **Alcohol (max. = 100)** |  |  |  |
| Beer taxation | Country with highest tax scores 15. The other countries’ scores are based on their tax rate as a percentage of the highest taxing country. | [Tax Foundation](https://taxfoundation.org/data/all/global/beer-taxes-in-europe-2023/) | 2023 |
| Wine taxation | Country with highest tax scores 20. The other countries’ scores are based on their tax rate as a percentage of the highest taxing country. | [Tax Foundation](https://taxfoundation.org/data/all/eu/wine-taxes-in-europe-2021/) | 2021 |
| Spirits taxation | Country with highest tax scores 25. The other countries’ scores are based on their tax rate as a percentage of the highest taxing country. | [Tax Foundation](https://taxfoundation.org/data/all/eu/distilled-spirits-taxes-europe-2021/) | 2021 |
| Tax incentives for low/ no alcohol content beer | If a country has implemented tax incentives for NoLo alcoholic beer it scores 10 points. | [WHO Alcohol Control Policies](https://www.who.int/data/gho/data/themes/topics/topic-details/GHO/alcohol-control-policies) | 2018 |
| Advertisement restriction national television | If a country differentiates between products, it scores 5 points. | [WHO Alcohol Control Policies](https://www.who.int/data/gho/data/themes/topics/topic-details/GHO/alcohol-control-policies) | 2018 |
| Advertisement restriction POS | If a country differentiates between products, it scores 5 points. | [WHO Alcohol Control Policies](https://www.who.int/data/gho/data/themes/topics/topic-details/GHO/alcohol-control-policies) | 2018 |
| Age limits on-premise service | If a country differentiates between products, it scores 5 points. | [WHO Alcohol Control Policies](https://www.who.int/data/gho/data/themes/topics/topic-details/GHO/alcohol-control-policies) | 2018 |
| Age limits off-premise service | If a country differentiates between products, it scores 5 points. | [WHO Alcohol Control Policies](https://www.who.int/data/gho/data/themes/topics/topic-details/GHO/alcohol-control-policies) | 2018 |
| Blood alcohol concentration (BAC) limit | Zero tolerance = 10 points <0.05% = 5 points 0.05% or above = 0 points | [WHO Alcohol Control Policies](https://www.who.int/data/gho/data/themes/topics/topic-details/GHO/alcohol-control-policies) | 2018 |
| **Sugar (max. = 100)** |  |  |  |
| Sugary food taxation | If a country taxes differently by sugar intake it scores 30 points. If a country levies an additional uniform excise tax on sugary food, it scores 15 points. | [European Commission: Implemented policies to address sugars intake](https://knowledge4policy.ec.europa.eu/health-promotion-knowledge-gateway/sugars-sweeteners-10_en) | 2021 |
| Sugar sweetened beverages (SSB) taxation | If a country taxes differently by sugar intake it scores 30 points. If a country levies an additional uniform excise tax on SSB it scores 15 points. | [European Commission: Implemented policies to address sugars intake](https://knowledge4policy.ec.europa.eu/health-promotion-knowledge-gateway/sugars-sweeteners-10_en) | 2022 |
| Nutrition labelling/ Traffic light | If a Nutriscore or something similar exists, a country scores 25 points. | [Eurofins: The Nutri-Score](https://www.eurofins.de/food-analysis/other-services/nutri-score/#:~:text=The%20Nutri%2DScore%20is%20currently,(keyword%3A%20multilingual%20labelling).) | 2024 |
| Youth advertisement | If a country restricts youth advertisement it scores 10 points. | [European Commission: Food and non-alcoholic beverage marketing to children and adolescents](https://knowledge4policy.ec.europa.eu/health-promotion-knowledge-gateway/sugars-sweeteners-10_en) | 2021 |
| Energy drinks restrictions | If specific regulations to energy drinks exist a country scores 5 points. | [Energy Drinks Europe: EU and self-regulation](https://www.energydrinkseurope.org/regulation/eu-and-self-regulation/);  [Nanny State Index](https://nannystateindex.org/wp-content/uploads/2023/05/NSI-2023.pdf) | Unclear |
| Vending machines restrictions | If specific regulations exist a country scores 5 points. | [EVA National Vending Associations](https://www.vending-europe.eu/wp-content/uploads/2018/05/Initiatives-to-encourage-a-wider-choice-products-in-vending-machines.pdf) | 2018 |

**Table A2.: Standardized Comparative Risk Evaluation Ranking**

|  | Tobacco ranking | | Alcohol ranking | | Sugar ranking | | **Total ranking** |
| --- | --- | --- | --- | --- | --- | --- | --- |
| UK | ***1*** | *4* | | ***3*** | | ***3*** | |
| Sweden | *4* | ***2*** | | *16* | | *7* | |
| Estonia | *10* | *7* | | *5* | | *7* | |
| Finland | *17* | ***1*** | | *5* | | *8* | |
| Switzerland | *6* | *13* | | *7* | | *9* | |
| Denmark | *8* | *6* | | *16* | | *10* | |
| Czech Republic | ***2*** | *16* | | *16* | | *11* | |
| France | *25* | *9* | | ***1*** | | *12* | |
| Belgium | *27* | *5* | | *4* | | *12* | |
| Greece | ***3*** | *10* | | *24* | | *12* | |
| Ireland | *27* | ***3*** | | *7* | | *12* | |
| Netherlands | *13* | *20* | | *7* | | *13* | |
| Slovakia | *7* | *17* | | *16* | | *13* | |
| Portugal | *12* | *25* | | *7* | | *15* | |
| Poland | *20* | *19* | | *7* | | *15* | |
| Lithuania | *23* | *8* | | *15* | | *15* | |
| Romania | *16* | *14* | | *16* | | *15* | |
| Spain | *24* | *21* | | ***2*** | | *16* | |
| Latvia | *22* | *11* | | *14* | | *16* | |
| Germany | *26* | *15* | | *7* | | *16* | |
| Slovenia | *21* | *12* | | *16* | | *16* | |
| Italy | *5* | *26* | | *24* | | *18* | |
| Luxembourg | *27* | *23* | | *7* | | *19* | |
| Croatia | *18* | *18* | | *24* | | *20* | |
| Cyprus | *9* | *27* | | *24* | | *20* | |
| Bulgaria | *14* | *30* | | *16* | | *20* | |
| Austria | *15* | *22* | | *24* | | *20* | |
| Serbia | *11* | *28* | | *24* | | *21* | |
| Malta | *27* | *23* | | *16* | | *22* | |
| Bosnia & Herzegovina | *19* | *28* | | *24* | | *24* | |

**Table A3.: Avoided Healthcare Costs (2023)**

| **Country** | **Possible avoided cost %** | **2023 health care costs (M€)** | **Avoided costs (M€)** |
| --- | --- | --- | --- |
| UK | 0,00% |  | 0,00 |
| Finland | 0,69% | 28.570,90 | 197,14 |
| Switzerland | 1,12% | 96.678,61 | 1.082,80 |
| Sweden | 1,16% | 60.357,79 | 700,15 |
| Denmark | 2,50% | 35.809,26 | 895,23 |
| Netherlands | 2,89% | 104.961,00 | 3.033,37 |
| France | 3,48% | 325.139,59 | 11.314,86 |
| Ireland | 3,92% | 33.531,30 | 1.314,43 |
| Austria | 3,92% | 52.778,85 | 2.068,93 |
| Germany | 4,10% | 491.563,00 | 20.154,08 |
| Luxembourg | 5,05% | 4.590,29 | 231,81 |
| Belgium | 5,10% | 64.412,05 | 3.285,01 |
| Spain | 5,21% | 138.155,33 | 7.197,89 |
| Italy | 6,19% | 179.156,00 | 11.089,76 |
| Czech Republic | 6,60% | 26.745,38 | 1.765,20 |
| Estonia | 6,67% | 2.852,94 | 190,29 |
| Portugal | 7,10% | 26.866,30 | 1.907,51 |
| Cyprus | 8,24% | 2.544,54 | 209,67 |
| Slovenia | 8,36% | 5.947,52 | 497,21 |
| Greece | 9,01% | 18.902,28 | 1.703,10 |
| Slovakia | 11,36% | 9.104,99 | 1.034,33 |
| Lithuania | 11,68% | 5.400,99 | 630,84 |
| Malta | 11,89% | 1.816,48 | 215,98 |
| Latvia | 12,12% | 2.865,46 | 347,29 |
| Poland | 15,05% | 53.676,56 | 8.078,32 |
| Croatia | 16,95% | 5.578,97 | 945,64 |
| Bulgaria | 22,18% | 7.499,47 | 1.663,38 |
| Romania | 22,88% | 18.518,25 | 4.236,98 |
| **Total** |  |  | **85.991,19** |

**Table A4.: SCORE Robustness Checks – Alternative Weighting and Normalization**

| **Country** | **Original SCORE (40/30/30)** | **Rank** | **Equal Weight (33.3%)** | **Rank** | **Z-score SCORE** | **Rank** |
| --- | --- | --- | --- | --- | --- | --- |
| UK | 58.10 | 1 | 56.44 | 1 | 68.20 | 1 |
| Finland | 53.07 | 2 | 53.37 | 2 | 65.09 | 2 |
| Sweden | 47.79 | 3 | 45.56 | 3 | 57.08 | 3 |
| Switzerland | 39.96 | 4 | 37.05 | 4 | 49.69 | 5 |
| Estonia | 38.69 | 5 | 36.93 | 5 | 49.77 | 4 |
| Spain | 35.58 | 6 | 35.11 | 6 | 48.67 | 6 |
| Czech Republic | 34.77 | 7 | 30.82 | 9 | 43.36 | 10 |
| Greece | 34.55 | 8 | 30.64 | 11 | 43.07 | 11 |
| Denmark | 34.28 | 9 | 31.46 | 8 | 44.02 | 8 |
| France | 33.23 | 10 | 34.55 | 7 | 48.51 | 7 |
| Netherlands | 32.80 | 11 | 30.72 | 10 | 43.89 | 9 |
| Portugal | 30.98 | 12 | 28.65 | 13 | 41.96 | 13 |
| Poland | 30.94 | 13 | 29.17 | 12 | 42.47 | 12 |
| Slovakia | 30.57 | 14 | 27.29 | 15 | 40.14 | 15 |
| Italy | 29.32 | 15 | 25.10 | 17 | 37.93 | 19 |
| Latvia | 28.80 | 16 | 27.51 | 14 | 40.84 | 14 |
| Romania | 27.62 | 17 | 25.08 | 18 | 38.13 | 18 |
| Lithuania | 26.80 | 18 | 25.34 | 16 | 38.57 | 17 |
| Cyprus | 24.99 | 19 | 21.42 | 22 | 34.56 | 24 |
| Slovenia | 24.45 | 20 | 22.56 | 20 | 35.85 | 20 |
| Croatia | 24.43 | 21 | 21.70 | 21 | 34.87 | 21 |
| Austria | 24.25 | 22 | 21.28 | 23 | 34.47 | 25 |
| Serbia | 23.17 | 23 | 19.73 | 27 | 33.01 | 27 |
| Bulgaria | 23.06 | 24 | 19.93 | 26 | 33.34 | 26 |
| Ireland | 21.48 | 25 | 24.98 | 19 | 38.90 | 16 |
| Bosnia & Herzegovina | 20.62 | 26 | 17.60 | 28 | 31.07 | 28 |
| Germany | 20.46 | 27 | 20.57 | 24 | 34.63 | 23 |
| Belgium | 17.08 | 28 | 20.09 | 25 | 34.74 | 22 |
| Luxembourg | 6.38 | 29 | 8.20 | 29 | 23.29 | 29 |
| Malta | 0.38 | 30 | 1.53 | 30 | 16.58 | 30 |

*Note*: Original SCORE uses 40/30/30 weighting (tobacco/alcohol/SSBs) with max-value normalisation within each domain. Equal Weight assigns 33.3% to each domain. Z-score applies z-score normalisation across countries within each domain, rescaled to 0–100, then applies 40/30/30 weights. Spearman rank correlation: Original vs. Equal Weight ρ = 0.97 (p < 0.001); Original vs. Z-score ρ = 0.94 (p < 0.001). Both alternative specifications yield substantively identical country rankings (Spearman ρ = 0.97 and ρ = 0.94, respectively), confirming that the composite index is robust to the weighting scheme and normalisation choice.
